# The role of long non-coding RNAs in NK cell biology and diseases

**DOI:** 10.1016/j.gendis.2025.101833

**Published:** 2025-08-25

**Authors:** Dan Zhang, Siqiao Wei, Qianqiu Wei, Zhansong Lin, Xiaoming Sun

**Affiliations:** a Zhejiang Key Laboratory of Medical Epigenetics, Department of Immunology and Pathogen Biology, School of Basic Medical Sciences, Hangzhou Normal University, Hangzhou, Zhejiang 311121, China; b State Key Laboratory for Diagnosis and Treatment of Infectious Diseases, The First Affiliated Hospital, College of Medicine, Zhejiang University, Hangzhou, Zhejiang 310003, China; c Laboratory of Integrative Cancer Immunology, Center for Cancer Research, National Cancer Institute, Bethesda, MD 20892, USA

**Keywords:** Cytotoxic activity, Innate immune cells, Long non-coding RNAs, Multiple diseases, NK cell

## Abstract

Long non-coding RNAs (lncRNAs) are pivotal regulators of gene expression, increasingly recognized for their roles in immune responses and disease progression. Natural killer (NK) cells, essential cytotoxic lymphocytes of the innate immune system, orchestrate immune responses through cytokine secretion and direct cytotoxicity. This review elucidates the immunomodulatory functions of lncRNAs in NK cell biology and their implications in pathological conditions. LncRNAs intricately govern key NK cell processes, including development, differentiation, activation, recruitment, cytotoxic function, and immune infiltration within the tumor microenvironment. These regulatory effects are mediated through diverse mechanisms, such as transcriptional control of effector molecules, miRNA sponging, metabolic reprogramming, protein ubiquitination, and epigenetic modifications. Focusing on NK cell infiltration in tumors, we classify lncRNAs into mechanistically defined and uncharacterized groups, highlighting their roles in tumor-associated competing endogenous RNA (ceRNA) networks, epigenetic regulation, and cell death pathways. By integrating these perspectives, this review enhances our understanding of lncRNA-mediated immune regulation and underscores their potential as therapeutic targets for diseases involving NK cell dysfunction.

## Introduction

Natural killer (NK) cells are crucial components of the innate immune system, capable of recognizing and eliminating infected cells or cancer cells through spontaneous cytotoxicity.^1^ The ability to distinguish between self and non/missing-self is acquired during the developmental stage by NK cells.^2^ A repertoire of diverse activating and inhibitory receptors is expressed on the surface of NK cells, and the activation of NK cells is regulated by the balance between these two types of receptors.^3^ Upon activation, mature NK cells perform their effector functions through the production of cytotoxic granules, death receptor ligands, and cytokines.^4^ Given their potent effector capabilities, NK cell-targeted immunotherapies have been extensively investigated across diverse pathological contexts, including malignancies, infectious diseases, autoimmune disorders, transplantation medicine, and aging-related diseases, revealing substantial therapeutic promise.^5^^,^^6^

Recent advances in whole-genome analytical tools, including high-throughput sequencing and microarrays, have enabled the systematic discovery of long non-coding RNAs (lncRNAs).^7^ LncRNAs are transcripts exceeding 200 nucleotides in length with no protein-coding potential or short peptides.^8^ The GENCODE database has systematically annotated over 20,000 lncRNA genes in the human genome, while emerging transcriptomic repositories suggest the potential existence of more than 100,000 lncRNA transcripts through advanced computational predictions expressed in a cell- or tissue-specific manner.^9^, ^10^, ^11^ LncRNAs are predominantly localized in the cytoplasmic and/or nuclear compartments, with their differential expression across distinct cell types underscoring their critical involvement in cellular molecular processes.^12^ These transcripts exhibit functional versatility across diverse biological processes, including protein biosynthesis, RNA maturation, and molecular signaling, act as decoy molecules or trafficking regulators, and mediate transcriptional gene silencing through chromatin structure modulation.^8^^,^^13^ Accumulating evidence has revealed their multifaceted roles in immune regulation, epigenetic programming, disease pathogenesis, and cellular differentiation.^14^^,^^15^ Despite the uncharacterized roles of many lncRNAs, their abundance suggests profound functional significance within the human transcriptome.

**Figure 1:**
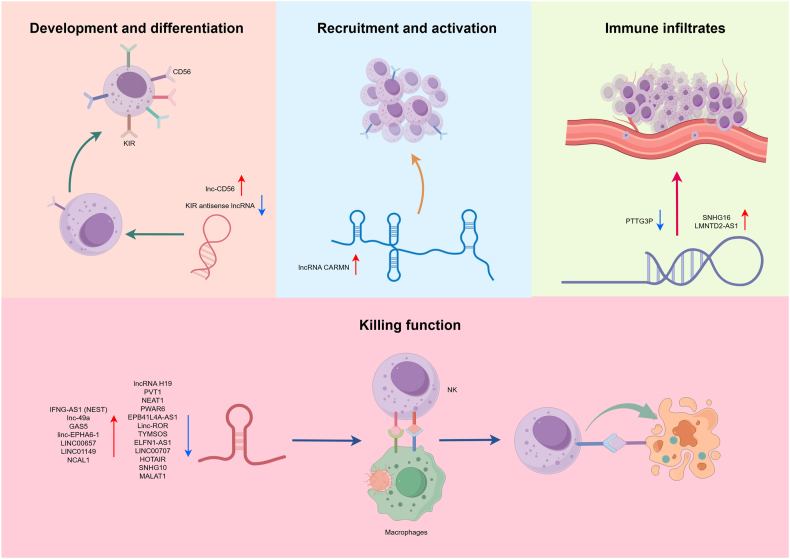
Overview of lncRNAs summarized in this study. The diagram shows lncRNAs and their role in the development, recruitment and activation, killing function and immune infiltration of NK cells. The red upward arrow represents positive regulation, the blue downward arrow represents negative regulation.

LncRNAs have emerged as critical regulatory molecules bridging a key gap in NK cell research, which has historically focused on receptor signaling, cytotoxic mechanisms, and transcription factor networks.^16^^,^^17^ Although lacking protein-coding capacity, certain lncRNAs potently modulate essential NK cell functions, including development, differentiation, migration, and cytotoxicity. Recent reviews have summarized the changes in the expression levels and mechanisms of action of relevant ncRNAs or lncRNAs in various pathophysiological processes from the perspective of immune cells, including innate immune cells and adaptive immune cells.^15^^,^^18^, ^19^, ^20^, ^21^, ^22^, ^23^ Some articles have also introduced ncRNAs affecting NK cell activity based on the classification of human systemic diseases.^23^, ^24^, ^25^ However, most of the above descriptions focus on miRNAs. In this review, we provide a comprehensive summary of lncRNAs in the immunoregulatory functions of NK cells (Fig. 1 and Table 1). We systematically catalog lncRNAs governing NK cell ontogeny, chemotactic recruitment, activation dynamics, cytotoxic effector mechanisms, and tumor immune infiltration programs, while elucidating their mechanistic underpinnings and therapeutic targetability. Further studies on the regulatory mechanisms and functional significance of these lncRNAs will deepen our understanding of NK cell biology and unlock novel immunotherapeutic strategies for diverse diseases.

**Table 1:** Summary of lncRNA type, location, pathways and functions of mechanistically characterized lncRNAs in NK cell regulation across various diseases.

NK cell development and differentiation							
lncRNA	LncRNA type	Species	Disease	RNA location	Affected signaling Molecules/Pathway	Function	Ref.
*KIR* antisense lncRNA	Antisense	H	Healthy	N/A	MZF-1/KIR signaling	Negative regulator of *KIR* gene expression early in development	28
lnc-CD56	Intronic, sense	H	Healthy	N/A	CD56 transcript	Positive regulator of CD56 expression	29

**NK killing**							

IFNG-AS1 (NEST)	Antisense	H	Healthy	Nucleus/Cytoplasm	IFNG-AS1/IFN-γ	Positive regulator of IFN-γ	49
NeST	Antisense	H	CD48^+^ tumors	Nucleus/Cytoplasm	NeST/IFN-γ	Positive regulator of IFN-γ	50
IFNG-AS1	Antisense	M	MCMV infection	Nucleus/Cytoplasm	IFNG-AS1/IFN-γ	Positive regulator of IFN-γ	51
lnc-49a	N/A	H	RSA	N/A	CD49a/perforin/IFN-γ/Granzyme B	Positive regulator of CD49a and cytotoxicity of dNK	52
LINC00707	Intergenic	H	HCC	Cytoplasm/Nucleus	YTHDF2 ubiquitination/TNF-α/IFN-γ	Negative regulator of NK cell antitumour activity	53
HOTAIR	Antisense	M	Leukemia	N/A	Wnt/β-catenin signaling	Negative regulator of IFN-γ	54
SNHG10	N/A	H	CRC	Exosome	INHBC/perforin/Granzyme B/NK1.1	Negative regulator of NK cell cytotoxicity	55
MALAT1	Intergenic	H	HBV infection	Nucleus/Cytoplasm	hsa-miR-155-5p/HIF-1α/IFN-γ	Negative regulator of IFN-γ transcription and secretion	57
GAS5	Sense	H	HCC	Nucleus/Cytoplasm	miR-544/RUNX3/IFN-γ/CD107a	Positive regulator of NK killing effect	58
GAS5	Sense	H	GC	Nucleus/Cytoplasm	miR-18a/IFN-γ/TNF-α	Positive regulator of NK killing effect	59
linc-EPHA6-1	Intergenic	H	Zika infection	Cytoplasm/Nucleus	hsa-miR-4485-5p/NKp46	Positive regulator of NK cytotoxicity	60
LINC00657	Intergenic	H	CC	N/A	miR-20a-5p/RUNX3/DR5	Positive regulator of NK cytotoxicity	61
MALAT-1	Intergenic	H	TNBC	Nucleus/Cytoplasm	miR-34a/MICA/B/perforin/Granzyme B/IFN-γ	Negative regulator of NK cell cytotoxicity	62
LINC01149	Intergenic	H	HCC	N/A	miR-128-3p/MICA	Positive regulator of NK recruitment and cytotoxicity	63
lncRNA H19	Intergenic	H	BC	Nucleus/Cytoplasm	miR-17-5p/STAT3/ULBP2	Negative regulator of NK cell cytotoxicity	64
PVT1	Intergenic	H	LSCC	Nucleus/Cytoplasm	miR-1301-3p/MBNL1	Negative regulator of NK cell cytotoxicity	65
NEAT1	Intergenic	M	Sepsis	Cytoplasm/Nucleus	miR-125/MCEMP1	Negative regulator of NK cell cytotoxicity	66
PWAR6	N/A	H	CRLM	Nucleus/Cytoplasm	Keap1/NRF2/SLC38A2	Negative regulator of NK cell function by altering glutamine availability	68
EPB41L4A-AS1	Antisense	H	NB	N/A	Encapsulated in exosomes and transmitted from CD56^bright^ NK to CD56^dim^ NK cells	Negative regulator of NK cell function by inhibiting glycolysis	70
Linc-ROR	Intergenic	H	GC	N/A	RXRA ubiquitination/MICB	Negative regulator of NK cell killing activity	72
TYMSOS	N/A	H	BC	Cytoplasm/Nucleus	CBX3/SYVN1/ULBP3 ubiquitination	Negative regulator of NK cell cytotoxicity	73
NCAL1	N/A	H	Healthy	Nucleus/Cytoplasm	Gab2/H3K4me3/H3K27/PI3K-AKT/CD107a	Positive regulator of NK cell cytotoxicity	74
ELFN1-AS1	Antisense	H	CRC	Cytoplasm/Nucleus	GDF15/JNK/NKG2D/GZMB	Negative regulator of NK cell NK surveillance and cytotoxicity	75

**NK cell infiltration**							

PTTG3P	N/A	H	CRC	Cytoplasm/Nucleus	HIF1α/PTTG3P/YAP1	Negatively correlates with NK cell infiltration	82
SNHG16	Intergenic	H	SKCM	N/A	hsa-let-7b-5p-TUBB4A	Positively correlates with NK cell infiltration	83
LMNTD2-AS1	Antisense	H	PCa	N/A	NRF2/FUS	Positively correlates with CD56^bright^ NK cell abundance	84

Footnotes:

LncRNA type: N/A, Not applicable; Species: H, Homo sapiens; M, Mice.

Disease: MCMV, murine cytomegalovirus; RSA, recurrent spontaneous abortion; HCC, hepatocellular carcinoma; CRC, colorectal cancer; HBV, hepatitis B virus; GC, gastric cancer; CC, cervical cancer; TNBC, triple negative breast cancer; BC, breast cancer; LSCC, laryngeal squamous cell carcinoma; CRLM, colorectal cancer liver metastases; NB, neuroblastoma; SKCM, skin cutaneous melanoma; PCa, prostate cancer.

## LncRNAs in NK cell development and differentiation

Emerging evidence highlights the critical roles of specific lncRNAs in modulating NK cell development and differentiation (Table 1). Killer cell immunoglobulin-like receptors (KIRs) are a critical family of receptors distributed on NK cell surfaces.^26^ They recognize HLA class I molecules bound with peptides presented on target cells and regulate NK cell activity through both inhibitory and activating functions.^3^ KIR genes remain completely silenced in NK progenitor cells to ensure that they do not negatively impact NK cell differentiation.^27^ A notable example of lncRNA involvement is the KIR antisense lncRNA, transcribed from the antisense orientation of KIR protein-coding genes and expressed exclusively in progenitor or pluripotent cell lineages.^28^ The distal KIR antisense lncRNA, expressed exclusively at the earliest stages of cell differentiation, enforces epigenetic silencing of KIR loci in NK progenitors to impede the NK cell differentiation process.^28^

In addition to their role in differentiation, lncRNAs also contribute to NK cell development, particularly in regulating maturation markers. For instance, the NK-specific lncRNA lnc-CD56 exhibits a strong positive correlation with the surface expression of CD56, a hallmark maturation marker of human NK cell differentiation and functional ontogeny.^16^^,^^29^ This implies that lnc-CD56 may function as a positive regulator of CD56 in primary human NK cells and differentiate NK cells from human CD34^+^ hematopoietic progenitor cells. Together, these findings illustrate how lncRNAs serve as critical molecular orchestrators in NK cell development and differentiation. By modulating epigenetic landscapes and transcriptional networks, lncRNAs such as KIR antisense lncRNAs and lnc-CD56 fine-tune the molecular events that govern NK cell ontogeny, offering novel insights into their regulatory potential and therapeutic relevance in NK cell-based immunotherapies.

## LncRNAs in NK cell recruitment and activation

During tumorigenesis, NK cells are recruited into the tumor microenvironment (TME) via chemokine signaling and achieve full activation following cancer cell recognition.^30^ Unlike cytotoxic T lymphocytes, which rely on T cell receptor signaling, the activation state of NK cells is controlled by a combination of activating and inhibiting receptor signal transduction.^31^^,^^32^ Several lncRNAs have been linked to the NK cell recruitment and activation in the context of carcinomas (Table 1). One prominent example is the lncRNA CARMN, whose expression positively correlates with the abundance of NK cells, T cells, and macrophages across various carcinomas.^33^ This correlation suggests that CARMN may enhance NK cell recruitment or activation within the TME, potentially preventing NK cell exhaustion by modulating interactions with macrophages.^33^ This suggests that CARMN may be a key regulator of the tumor immune microenvironment, with potential implications for immune activation, therapeutic targeting, and biomarker development in multiple cancer types.^33^ Similarly, computational analyses have identified other lncRNAs with distinct roles in NK cell dynamics. For instance, N^6^-methyladenosine (m^6^A)-related lncRNAs in bladder cancer show a positive correlation with resting NK cells, whereas N^7^-methylguanosine-related lncRNAs in colon adenocarcinoma and specific TF–mRNA–lncRNA networks in atopic dermatitis are negatively associated with resting NK cell populations.^34^, ^35^, ^36^ These differential associations highlight the context-specific roles of lncRNAs in modulating NK cell states. Furthermore, the establishment of an immune-related lncRNA (IRL) classifier has enabled precise predictions of prognosis and immunotherapy responsiveness in patients with laryngeal squamous cell carcinoma (LSCC).^37^ Consistent with computational predictions, this model demonstrated that low-risk patient cohorts exhibit significant enrichment of activated NK cells within the TME, underscoring the potential of lncRNAs to shape immune infiltration and effector function (29). Despite these promising insights, current studies primarily document correlative relationships between lncRNAs and NK cell recruitment or activation, with limited exploration of the underlying molecular mechanisms.^37^

## LncRNAs in the killing function of NK cells

NK cells mediate target cell lysis through coordinated mechanisms involving perforin/granzyme exocytosis and Fas/Fas ligand (FasL) death receptor interactions, while simultaneously executing antibody-dependent cellular cytotoxicity (ADCC). Additionally, NK cells secrete immunomodulatory cytokines, such as IFN-γ, to orchestrate multifaceted cytotoxic responses.^38^ Bioinformatics approaches, integrating differential gene expression profiling and functional enrichment analysis, have systematically identified lncRNAs associated with NK cell cytotoxicity in diverse disease models.^39^, ^40^, ^41^, ^42^, ^43^, ^44^, ^45^, ^46^, ^47^ These lncRNAs play both autonomous regulatory roles and cooperative interactions within competing endogenous RNA (ceRNA) networks, engaging microRNAs, circular RNAs, and protein-coding transcripts. These interactions position lncRNAs as promising theranostic biomarkers within multi-omics frameworks for biomarker discovery.^39^, ^40^, ^41^, ^42^, ^43^, ^44^, ^45^, ^46^, ^47^ Mechanistically, lncRNAs modulate NK cell killing functions through diverse pathways, including transcriptional regulation of cytotoxicity-related genes, miRNA sponging, metabolic reprogramming, protein ubiquitination, and epigenetic modifications (Table 1). These multifaceted regulatory roles underscore the complexity of lncRNA-mediated control over NK cell cytotoxicity.

## LncRNA-driven transcriptional regulation of cytolytic effectors

NK cells eliminate target cells through the secretion of cytotoxic mediators, including IFN-γ, granzyme B, and perforin.^48^ A well-documented example is lncRNA IFNG-AS1 (also known as NEST), which is rapidly induced upon NK cell activation and enhances IFN-γ secretion when overexpressed.^49^ Similarly, in a murine immunotherapy combining NK cell-mediated tumor targeting and lncRNA, lncRNA NeST overexpression in YTS cells was observed to enhance interferon secretion upon encountering CD48^+^ tumor cells, thereby augmenting cytotoxic efficacy.^50^ Conversely, impaired lncRNA regulation can compromise NK cell maturation and function. For instance, in neonatal mice infected with murine cytomegalovirus (MCMV), downregulated expression of lncRNA IFNG-AS1 in NK cells severely reduces IFN-γ production, leading to defective maturation and diminished cytotoxic capacity.^51^ These findings highlight the critical role of lncRNAs in sustaining IFN-γ-mediated effector functions. Beyond IFN-γ, lncRNAs regulate other cytotoxic mediators in NK cells. For example, lnc-49a acts as a positive regulator of CD49a in primary human decidual NK cells from patients with recurrent spontaneous abortions. Knockdown of lnc-49a via siRNA significantly reduces the production of perforin, IFN-γ, and granzyme B, underscoring its role in maintaining NK cell cytotoxicity.^52^ Similarly, LINC00707 enhances the cytotoxicity of NK-92MI cells against hepatocellular carcinoma (HCC) cells by interacting with YTH N6-methyladenosine RNA-binding protein 2 (YTHDF2).^53^ In contrast, certain lncRNAs exert immunosuppressive effects. Upregulation of the lncRNA HOTAIR through mimic transfection in murine models reduces IFN-γ levels in peripheral blood and diminishes NK cell activity by activating the Wnt/β-catenin signaling pathway, thereby promoting immune evasion in leukemia.^54^ Likewise, the exosomal lncRNA SNHG10, which is up-regulated in colorectal cancer cells, suppresses perforin, granzyme B, and NK1.1 expression in NK cells by activating the TGF-β signaling pathway via inhibin subunit beta C (INHBC), facilitating tumor immune escape.^55^

## LncRNAs as ceRNAs modulating NK cytotoxicity

**Figure 2:**
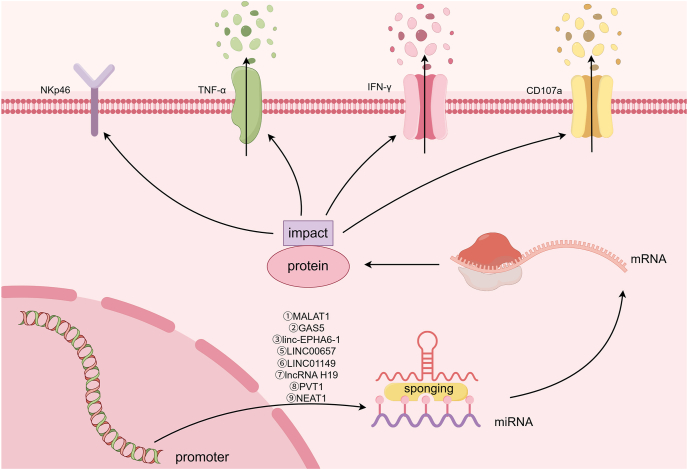
LncRNAs as ceRNA. LncRNAs act as miRNA sponges to regulate NK cell toxicity.

Certain lncRNAs can function as “miRNA sponges” through competitive miRNA binding. Consequently, these lncRNAs located on NK cells act as ceRNAs to regulate the function of miRNAs and thus affect NK cytotoxicity^56^ (Fig. 2 and Table 1). For example, in NK cells from tenofovir-treated pregnant women with hepatitis B virus infection, lncRNA MALAT1 expression is significantly down-regulated, which consequently suppresses IFN-γ transcription and production.^57^ Mechanistically, MALAT1 functions as a ceRNA by sponging hsa-miR-155-5p to up-regulate HIF-1a, promoting NK cell immune recovery.^57^ Similarly, the lncRNA GAS5 is significantly downregulated in NK cells from HCC patients.^58^ The overexpression of GAS5 enhances RUNX3 expression, IFN-γ secretion, and NK cell cytotoxic activity by targeting the miR-544/RUNX3 axis, which is an effect reversed by miR-544 mimic.^58^ In gastric cancer (GC), NK cells show decreased lncRNA GAS5 and increased miR-18a expression. GAS5 deficiency suppresses IFN-γ/TNF-α secretion and cytotoxicity in NK cells, while miR-18a inhibition rescues cytotoxic activity, indicating that GAS5 enhances NK-mediated antitumor effects by sponging miR-18a.^59^ Additionally, linc-EPHA6-1 in IFNβ-induced exosomes enhances NK cell cytolytic activity by sponging hsa-miR-4485-5p, up-regulating the natural cytotoxicity receptor NKp46 in both NK92 cells and primary NK cells.^60^

In addition to acting as ceRNAs in NK cells, there are several lncRNAs located in tumor cells or T cells that can regulate the killing toxicity of NK cells to target cells. In cervical cancer (CC), miR-20a-5p was confirmed as a target of LINC00657. LINC00657 has been demonstrated to promote the cytotoxic activity of NK cells by inducing the miR-20a-5p/RUNX3/DR5 axis, where DR5 is a downstream target of RUNX3.^61^ In triple negative breast cancer (TNBC) patients, the up-regulated lncRNA MALAT-1 inhibits NK cell cytotoxicity, but silencing MALAT-1 enhances NK cell activity, potentially via the miR-34a or miR-17-5p pathway.^62^ In HCC, the LINC01149 variant rs2844512 acts as a ceRNA by sponging miR-128-3p to induce MHC class I chain-related protein A (MICA) expression, which recruits NK cells.^63^ While this mechanism potentially recruits NK cells to lyse infected cells, concomitant shedding of highly soluble MICA paradoxically promotes NK cell exhaustion and tumor immune evasion through sustained receptor–ligand interactions.^63^ In young breast cancer (BC) patients, lncRNA H19 functions as a ceRNA by sequestering miR-17-5p, thereby counteracting its suppressive activity and blocking the miR-17-5p-mediated down-regulation of STAT3.^64^ The ectopic expression of miR-17-5p in MDA-MB-231 cells significantly inhibited the expression levels of STAT3 and lncRNA H19, and induced the expression of ULBP2, a NKG2D ligand, in TNBC cell lines.^64^ In LSCC, the up-regulated lncRNA, PVT1, promotes tumor cell proliferation and resistance to NK cell lysis by sponging miR-1301-3p to up-regulate MBNL1.^65^ Similarly, in T cells from sepsis patients, the lncRNA NEAT1 acts as a ceRNA for miR-125 to up-regulate its target gene MCEMP1, inhibiting NK cell activity and accelerating the pathological progression of sepsis.^66^

## LncRNAs orchestrate metabolic reprogramming in NK cell cytotoxicity

**Figure 3:**
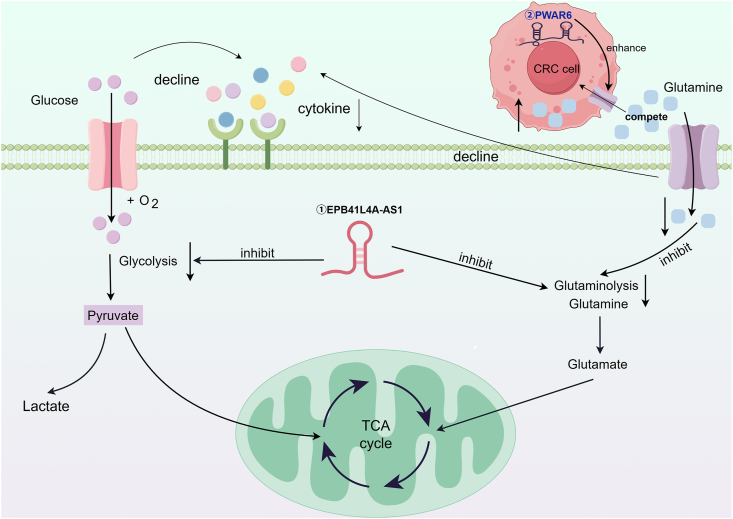
LncRNAs orchestrate metabolic reprogramming. LncRNAs regulate the killing function of NK cells by participating in the tricarboxylic acid cycle and glucose glycolysis.

LncRNAs modulate NK cell cytotoxicity through diverse mechanisms, with metabolic reprogramming emerging as a critical, yet underexplored, regulatory pathway (Fig. 3 and Table 1). Metabolic reprogramming critically drives tumor progression and metastasis, with glutamine serving as a vital substrate for the tricarboxylic acid (TCA) cycle and precursor for lipid, nucleotide, and hexosamine biosynthesis.^67^ In colorectal cancer (CRC) liver metastases, exosomal lncRNA PWAR6, derived from myofibroblastic cancer-associated fibroblasts (myCAFs), is markedly up-regulated.^68^ PWAR6 competes with Keap1 to stabilize NRF2 and up-regulate SLC38A2 expression, thereby increasing glutamine uptake in CRC cells.^68^ This metabolic competition depletes extracellular glutamine, a critical resource for NK cell activation, resulting in impaired cytotoxicity due to substrate deprivation.^68^ Glucose metabolism also serves as a primary bioenergetic source for NK cell activation.^69^ Similarly, in neuroblastoma, NK cells exhibit glycolytic arrest, which is mediated by the transfer of the elevated exosome lncRNA EPB41L4A-AS1 from the CD56^bright^ NK subset to CD56^dim^ NK subset.^70^

## LncRNA-mediated control of ubiquitination in NK cell cytotoxicity

**Figure 4:**
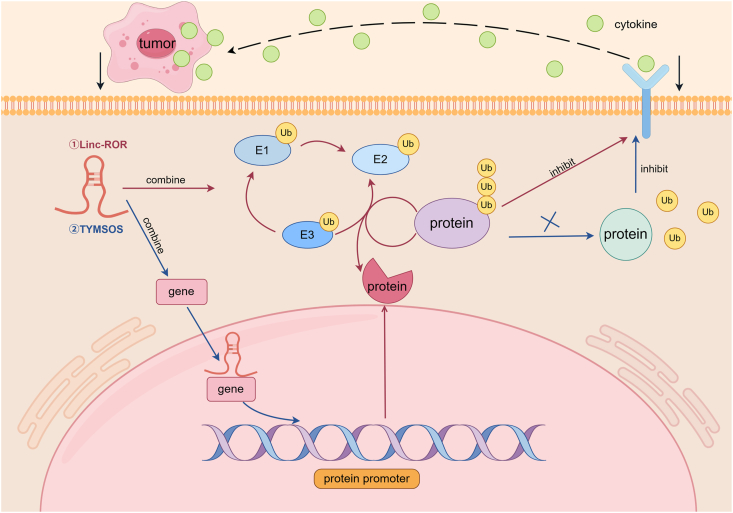
LncRNA-mediated control of ubiquitination. Linc-ROR and the lncRNA TYMSOS alter the cytotoxicity of NK cells against tumor cells by participating in protein ubiquitination and degradation. The red line indicates the linc-ROR pathway, and the blue line indicates the TYMSOS pathway.

Ubiquitination is a process of post-translational modification that degrades target proteins and facilitates protein interactions, cell localization, and signal transduction.^71^ LncRNAs have also been found to influence NK cytotoxicity by regulating ubiquitination (Fig. 4 and Table 1). For instance, the linc-ROR promotes the ubiquitination and degradation of retinoid X receptor alpha (RXRA) in gastric cancer cells, leading to reduced MHC class I chain-related protein B (MICB) expression and thereby suppressing NK cell cytotoxic activity in gastric cancer patients.^72^ Similarly, in breast cancer cells, the lncRNA TYMSOS promotes chromobox protein homolog 3 (CBX3)-mediated transcriptional repression and synoviolin 1 (SYVN1)-driven ubiquitin‒proteasomal degradation of UL16 binding protein 3 (ULBP3), suppressing NK cell cytotoxicity to facilitate tumor metastasis and immune escape.^73^ These findings highlight the critical role of lncRNAs in fine-tuning ubiquitination to modulate NK cell function and tumor progression.

## LncRNA-mediated epigenetic regulation in NK cell cytotoxicity

**Figure 5:**
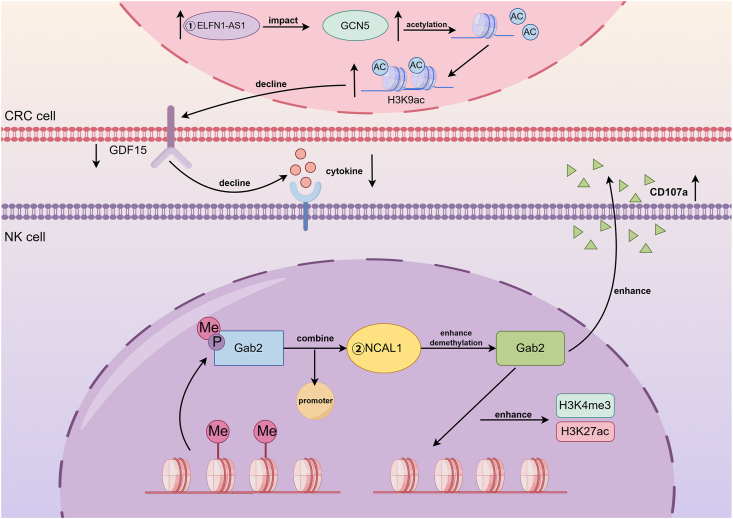
LncRNA-mediated epigenetic regulation. The LncRNAs NCAL1 and ELFN1-AS1 affect NK cell toxicity by regulating epigenetic modifications and the PI3K-AKT and GDF15/JNK signaling pathways.

In NK cells, the nuclear lncRNA NCAL1 epigenetically regulates NK cell function by binding to the Gab2 promoter, reducing its methylation while increasing H3K4me3 and H3K27 levels, thereby enhancing NK cell cytotoxicity against tumor cells via the PI3K-AKT pathway.^74^ Conversely, the lncRNA ELFN1-AS1 enhances the ability of CRC cells to evade NK cell surveillance both *in vitro* and *in vivo*.^75^ Mechanistically, ELFN1-AS1 down-regulates NKG2D and GZMB expression via the GDF15/JNK pathway. Concurrently, it strengthens the interaction between the GCN5 and SND1 proteins, reducing H3K9ac enrichment at the GDF15 promoter to up-regulate GDF15 production in CRC cells.^75^ Collectively, these studies provide compelling evidence for the regulatory role of lncRNAs in NK function via epigenetic regulation (Fig. 5 and Table 1).

## LncRNA regulates NK cell infiltration in the TME

The TME is a complex system comprising diverse cell types, cytokines, and extracellular components.^76^, ^77^, ^78^ The type and abundance of tumor-infiltrating immune cells critically influence tumor progression.^79^^,^^80^ Specific lncRNAs critically regulate NK cell infiltration, influencing tumor immune dynamics via diverse mechanisms.

## Mechanistically defined lncRNAs in NK immune infiltration

In colorectal cancer, the lncRNA PTTG3P enhances tumor cell proliferation and glycolysis via the HIF1α/PTTG3P/YAP1 axis and exhibits a negative correlation with NK, CD8 T, and other immune cell infiltrations.^81^ Similarly, the lncRNA SNHG16 in skin cutaneous melanoma (SKCM) regulates the hsa-let-7b-5p-TUBB4A axis, potentially linked to NK cell immune infiltration.^82^ In prostate cancer patients, the lncRNA LMNTD2-AS1 is the only lncRNA associated with both overall survival (OS) and progression-free interval (PFI).^83^ Its elevated expression correlates positively with CD56^bright^ NK cell abundance by regulating the NRF2 signaling pathway through targeting FUS.^83^

## Mechanistically unexplored lncRNAs in NK immune infiltration

Furthermore, although certain lncRNAs have been shown to be significantly associated with NK cell infiltration in the TME, their mechanisms remain unexplored. In this section, we summarized lncRNAs associated with CD56^bright^, CD56^dim^, or total NK cell populations across diverse tumor microenvironments. Although mechanistic elucidation is lacking, these findings suggest functional disparities of such lncRNAs within distinct NK cell subsets within the tumor microenvironment. In lung adenocarcinoma (LUAD), the expression of the lncRNA NPSR1-AS1 was negatively associated with CD56^dim^ NK cells but positively correlated with CD56^bright^ NK cells, suggesting that NPSR1-AS1 may have different functions in different NK subgroups.^84^ Similarly, lncRNA ITGB1-DT levels positively correlate with CD56^dim^ NK cell abundance, suggesting its potential role in modulating NK cell infiltration.^85^ The expression of the LncRNA HAND2-AS1 positively correlates with NK cell infiltration in the TME, a pan-cancer association suggesting its potential as a tumor suppressor.^86^ Conversely, the lncRNA PRR7-AS1 inversely correlates with CD56^bright^ NK cell abundance and serves as a prognostic biomarker.^87^ In gastric cancer patients with peritoneal metastasis, elevated LINC00924 expression predicts poor survival and increased NK cell infiltration.^88^ In HCC patients, the lncRNA INKA2-AS1 is recognized as an independent prognostic factor for overall survival.^89^ Its expression positively correlates with immune cells such as CD56^bright^ NK cells, helper T cells, Th2 cells, and macrophages, suggesting its role as a critical immune response modulator in HCC.^89^ In cholangiocarcinoma (CCA), the expression of the lncRNA AL161431.1 is significantly up-regulated.^90^ Its high expression positively correlates with increased infiltration of CD56^dim^ NK cells, T cells, and helper T cells, while AL161431.1 knockdown in CCA cells suppresses tumor cell invasion, migration, and proliferation, highlighting its potential as a therapeutic target for CCA.^90^ In addition, LINC01589 in uterine corpus endometrial carcinoma (UCEC)^91^ and MSC-AS1 in gastric cancer^92^ are positively related to NK cell abundance, while the lncRNA MIAT in breast cancer (BC),^93^ the lncRNA ZFHX4-AS1 in ovarian cancer,^94^ and PCBP1-AS1 in cervical cancer^95^ are negatively related to NK cell abundance, suggesting diverse roles in NK cell modulation. The discovery of lncRNAs linked to NK cell infiltration opens new possibilities for current conventional therapies, but mechanistic studies need to be further explored.

## NK infiltration-associated lncRNAs in tumor ceRNA networks

Bioinformatics analyses have identified additional lncRNAs potentially linked to NK cell infiltration, though experimental validation or mechanistic exploration remains lacking. Notably, ceRNA networks, particularly lncRNA-miRNA-mRNA regulatory axes, are increasingly implicated in tumorigenesis and prognosis across cancers, offering frameworks for dissecting oncogenic mechanisms.^96^ Differentially expressed lncRNAs (DElncRNAs) were identified through computational screening using algorithms like CIBERSORT. Intriguingly, DElncRNAs in radiation-induced esophageal injury,^97^ hepatocellular carcinoma,^98^ and thyroid cancer^99^ showed positive correlations with NK cell infiltration levels, whereas those in idiopathic pulmonary fibrosis^100^ and diabetic kidney disease^101^ exhibited negative correlations.

## Epigenetic modification-linked lncRNAs in the TME

The main RNA methylation modifications, including m^6^A, 5-methylcytosine (m^5^C), and N1-methyladenosine (m^1^A) in mRNAs and ncRNAs, play critical regulatory roles in tumor RNA modifications.^102^^,^^103^ In gastric cancer, m^6^A-related lncRNAs were identified to construct a risk model stratifying patients into high- and low-risk groups. The risk score demonstrated negative correlations with activated NK cells^104^ or with resting NK cells^105^ across studies. In colorectal cancer,^105^ bladder cancer,^106^^,^^107^ and pancreatic ductal adenocarcinoma,^108^ tumor immune profiling of risk models constructed from m^6^A-related lncRNAs revealed significantly elevated resting NK cell infiltration levels in high-risk groups. In lung squamous cell carcinoma (LUSC), prognostic models based on m^5^C-related lncRNAs showed a negative correlation between risk scores and activated NK cells,^109^ whereas in head and neck squamous cell carcinoma (HNSCC), m^6^A/m^5^C/m^1^A-related lncRNA models positively correlate with resting NK cells.^110^ These findings highlight the significance of epigenetic modification-associated lncRNAs in tumor prognosis and NK cell infiltration, providing a framework for further functional and mechanistic exploration.

## Cell death mechanism-linked lncRNAs in tumors

Cuproptosis, ferroptosis, and pyroptosis are emerging as novel regulatory cell death mechanisms, with mounting evidence linking them to tumorigenesis and cancer progression.^111^, ^112^, ^113^ Cuproptosis-related lncRNA risk models in HCC, gastric cancer, colon cancer, HNSCC, and ovarian serous cystadenocarcinoma show negative correlations with NK cell activation.^114^, ^115^, ^116^, ^117^, ^118^ In gliomas, patients stratified by ferroptosis-related lncRNA expression into high- and low-risk groups showed elevated levels of both activated and resting NK cells in high-risk cohorts.^119^ In contrast, stratification based on pyroptosis-related lncRNAs revealed a positive correlation between risk score and activated NK cells only.^120^ In Wilms tumor, ferroptosis-related lncRNAs were identified and used for patient stratification. Analysis revealed significantly elevated tumor NK cell levels in high-risk groups.^121^ These findings suggest that lncRNAs linked to cell death-regulatory mechanisms may serve as promising molecular therapeutic targets.

## Bioinformatics identified lncRNAs associated with NK infiltration

Furthermore, lncRNAs are implicated across multiple cancer types, where they correlate with the infiltration of activated or resting NK cells in the tumor immune microenvironment and serve as prognostic and diagnostic biomarkers.^85^^,^^122^, ^123^, ^124^, ^125^, ^126^, ^127^, ^128^, ^129^, ^130^, ^131^, ^132^, ^133^, ^134^, ^135^, ^136^, ^137^, ^138^, ^139^ Shao et al constructed a pan-cancer immune co-regulatory network of lncRNAs, revealing that these immune-cooperative lncRNAs (IC-lncRNAs) are predominantly enriched in immune cell populations and exhibit significant correlations with heterogeneous immune infiltration patterns, thereby uncovering conserved immunomodulatory mechanisms across malignancies.^140^ These bioinformatics-derived findings necessitate further functional dissection, particularly exploration of context-specific molecular mechanisms, to advance the mechanistic understanding of disease pathogenesis.

## Therapeutic strategies targeting lncRNAs in NK cells

The clinical significance of lncRNA dysregulation is underscored by its potential as diagnostic biomarkers and therapeutic targets.^141^ For example, urinary lncRNA PCA3 is an FDA-approved biomarker for prostate cancer diagnosis.^142^ In the H19-targeted therapy program for bladder cancer (NCT04234216), intravesical administration of BC-819 achieves maximal local exposure to targeted bladder cancer cells, and the program has now entered Phase II clinical trials.^143^ Class inhibitors of the lncRNA MALAT1-FTX-001 are undergoing preclinical validation, with potential progression to Phase I trials upon safety clearance.^144^ As previously mentioned, MALAT-1 functions as a ceRNA to regulate NK cell cytotoxicity, highlighting its significant therapeutic potential in NK cells. Other lncRNA-targeted strategies, including those against HOTAIR and NEAT1, are in preclinical testing.^145^^,^^146^ Upon optimization of their stability and targeted delivery technologies, therapies based on these lncRNAs are poised to enter clinical trials. Considering HOTAIR’s transcriptional regulation of cytotoxic factors and NEAT1’s role as a ceRNA in modulating NK cell cytotoxicity, further mechanistic exploration will help address the gap in targeted therapies for immune-related lncRNAs across multiple diseases.

## Conclusion and future perspectives

Growing evidence highlights the critical roles of lncRNAs as regulators of NK cell biology. LncRNAs regulate the levels of translatable mRNAs in response to specific developmental or stimulus cues, which provides the possibility of controlling the cytotoxicity and cytokine secretion of NK cells. These findings deepen our understanding of immune regulation and its implications in diverse diseases. In this review, we comprehensively summarize the reported lncRNAs and their functions across various diseases, focusing on four key aspects of NK cell biology: development/differentiation, recruitment/activation, killing function, and tumor infiltration. Notably, lncRNAs regulate the transcription of NK cell cytotoxic molecules through direct or indirect mechanisms, with their role as miRNA sponges being widely studied. This underscores the complexity of lncRNA-mediated immune regulation.

**Figure 6:**
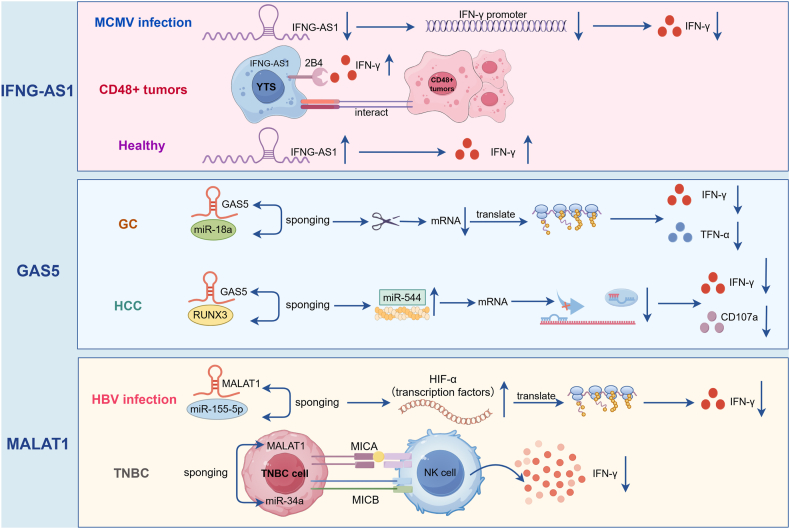
Common lncRNAs in different diseases. Some lncRNAs affect the function of NK cells through different pathways, such as IFNG-AS1 (NEST) in healthy individuals, CD48^+^ tumors, and MCMV infections; GAS5 in HCC and GC; and MALAT-1 in HBV infection and TNBC.

To date, numerous lncRNA-related studies in the immune system have focused on characterizing lncRNA functions in murine and human primary cells or cell lines. Nevertheless, emerging evidence has demonstrated that specific lncRNAs influence disease pathogenesis and progression across diverse pathologies by modulating NK cell activity, such as IFNG-AS1 in healthy individuals, CD48^+^ tumors, and MCMV infection; GAS5 in HCC and GC; MALAT1 in HBV infection and TNBC. All of these play pivotal roles in mediating protection against or susceptibility to inflammatory diseases by regulating NK cell functions. The same lncRNA has been proven to adopt different molecular mechanisms in different diseases (Fig. 6).

Nevertheless, the mechanisms of action of these lncRNAs remain incompletely elucidated. As highlighted in this review, numerous functional lncRNAs with potential diagnostic and prognostic utility have been identified. Future research should prioritize comprehensive mechanistic studies to uncover how lncRNAs govern NK cell functionality from multiple perspectives. Such efforts will strengthen their potential as innovative therapeutic targets, paving the way for novel immunotherapies in cancer and beyond.

## CRediT authorship contribution statement

**Dan Zhang:** Writing – review & editing, Writing – original draft, Visualization, Methodology, Investigation, Funding acquisition, Formal analysis, Data curation. **Siqiao Wei:** Software, Investigation. **Qianqiu Wei:** Methodology, Investigation. **Zhansong Lin:** Writing – review & editing, Supervision. **Xiaoming Sun:** Writing – review & editing, Funding acquisition, Conceptualization.

## Conflict of interests

The authors declare no conflicts of interest in any form.
